# Early experience with Watson for oncology in Korean patients with colorectal cancer

**DOI:** 10.1371/journal.pone.0213640

**Published:** 2019-03-25

**Authors:** Eui Joo Kim, Hyun Sun Woo, Jae Hee Cho, Sun Jin Sym, Jeong-Heum Baek, Won-Suk Lee, Kwang An Kwon, Kyoung Oh Kim, Jun-Won Chung, Dong Kyun Park, Yoon Jae Kim

**Affiliations:** 1 Division of Gastroenterology, Department of Internal Medicine, Gachon University Gil Medcal Center, Gachon University College of Medicine, Incheon, Republic of Korea; 2 Division of Oncology, Department of Internal Medicine, Gachon University Gil Medical Center, Gachon University College of Medicine, Incheon, Republic of Korea; 3 Department of Surgery, Gachon University Gil Medical Center, Gachon University College of Medicine, Incheon, Republic of Korea; University of Pennsylvania, UNITED STATES

## Abstract

**Background:**

Watson for oncology (WFO) is a cognitive computing system providing decision support. We evaluated the concordance rates between the treatment options determined by WFO and those determined by a multidisciplinary team (MDT).

**Methods:**

We reviewed the medical charts of patients diagnosed with colorectal cancer who visited the MDT at a single tertiary medical center from November 2016 to April 2017. WFO classified the treatment options for specific patients into three categories: ‘Recommended’, ‘For consideration’, and ‘Not recommended’. Concordance rates between the WFO- and MDT-determined chemotherapy options, and the factors that potentially influence the concordance rate, were analyzed.

**Results:**

Sixty-nine patients with colorectal cancer met with the MDT from Nov. 2016 to Feb. 2017. The mean age of the patients was 62 years (range: 34–86 years), and more patients were male (47/69) than female. Of the 69 patients, 51 (73.9%) were diagnosed with colon cancer, of whom 46.4% received the same regimen recommendation from WFO (‘Recommended’) as they did from the MDT. After inclusion of the ‘For consideration’ category from WFO, the concordance rate increased to 87.0%. The concordance rate between MDT and NCCN guidelines was 97.1%, and that between the WFO and NCCN guidelines was 88.4%. The concordance rates between WFO and MDT were significantly lower in patients with stage II, IIIC, or IV disease (P<0.001), and the colorectal cancer stage was the only statistically significant factor discriminating between WFO and MDT.

**Conclusions:**

The concordance rate between chemotherapy regimens for colorectal cancer determined by MDT versus WFO recommendations was 46.4%. After including the ‘For consideration’ category from WFO, the concordance rate increased to 88.4%. Further modification and improvement of the WFO prioritizing algorithm used to recommend treatment may increase the usefulness of WFO in the clinic.

## Introduction

Colorectal cancer is one of the most commonly diagnosed cancers, being the third most common in males and the second most common in females according to global cancer statistics [1]. Presently, chemotherapy options for patients with colorectal cancer are determined by multidisciplinary teams (MDTs) based on the National Comprehensive Cancer Network (NCCN) guidelines, recent studies in the literature, and clinical experience.

In October 2016, the National Academies of Sciences, Engineering, and Medicine held a meeting to discuss the manufacturing, social, and economic implications of the Fourth Industrial Revolution [2]. One area with implications for manufacturing is artificial intelligence (AI), which refers to the creation of intelligent machines that function and react similarly to humans [3]. For the application of AI in healthcare, some researchers have devised algorithms based on five real-life databases for breast cancer, colon cancer, diabetes, thyroid disease, and fetal heartbeat disorders [4]. In oncology, IBM developed Watson for oncology (WFO), an AI cognitive computing system that can suggest proper chemotherapy regimens for specific cancer patients.

WFO helps reduce the time needed to identify important information in a patient’s medical records, integrate relevant literature articles, and explore chemotherapy options. However, because cognitive computing technology was developed recently, data on its utility in clinical oncology are lacking. The aim of this study was to analyze the concordance rates between chemotherapy options determined by WFO versus MDTs.

## Patients and methods

The Institutional Review Board of Gachon University Gil Medical Center (GBIRB2017-292) approved the study protocol. Between November 2016 and April 2017, 69 patients who were candidates for systemic chemotherapy to treat colorectal cancer received multidisciplinary care services, including WFO. Chemotherapy included adjuvant, neoadjuvant, and palliative treatment. Among these patients, 45 underwent surgical treatment before multidisciplinary services, 7 needed chemotherapy for palliation, and the remaining had a history of other chemotherapeutic treatments (Table 1).

**Table 1:** Baseline clinical characteristics of the patients.

	Patients who participated in Watson for oncology (n = 69)
Age, mean ± SD	62.30 ± 12.44
Male, n (%)	47 (68.1)
Primary tumor location, n (%)	
Colon	51 (73.9%)
Rectum	18 (26.1%)
ECOG performance status	
0	5 (7.2%)
1	64 (92.8%)
Prior therapy, n (%)	
Surgery	45 (65.2%)
Adjuvant chemotherapy	10 (14.5%)
Postoperative CCRT	6 (8.7%)
Neoadjuvant CCRT	1 (1.4%)
No prior treatment	7 (10.1%)
Stage, n (%)	
I	1 (1.4%)
IIA	10 (14.5%)
IIB	1 (1.4%)
IIIA	4 (5.8%)
IIIB	27 (39.1%)
IIIC	3 (4.3%)
IV	23 (33.3%)
Ras mutation, n (%)	26 (37.7%)
EGFR mutation, n (%)	51 (73.9%)
Recurrence, n (%)	8 (11.6%)

CCRT, concurrent chemoradiotherapy; EGFR, epidermal growth factor receptor

### Multidisciplinary team

The MDT is comprised of oncologists, gastroenterologists, surgeons, and radiologists who discuss the advantages and disadvantages of each candidate chemotherapy regimen. The MDT prioritized the chemotherapy regimens for patients, and the MDT made the final decision. Independently of the MDT’s decision, WFO also suggested therapeutic options for the same patient. The MDT explained to the patients the prioritization of the chemotherapy options, as determined by their team and WFO, as well as their reason for the final therapeutic decision.

### Watson for oncology

During the study period, IBM WFO version 16.9 was used. To obtain chemotherapy options using WFO, the MDT inputted the information of the 69 patients into the system, including age, sex, past medical history, stage, previous chemotherapy, resectability of metastatic lesions, Ras and EGFR mutation statuses, lymphovascular invasion, number of lymph nodes examined, neural invasion, and MSI status.

**Fig 1:**
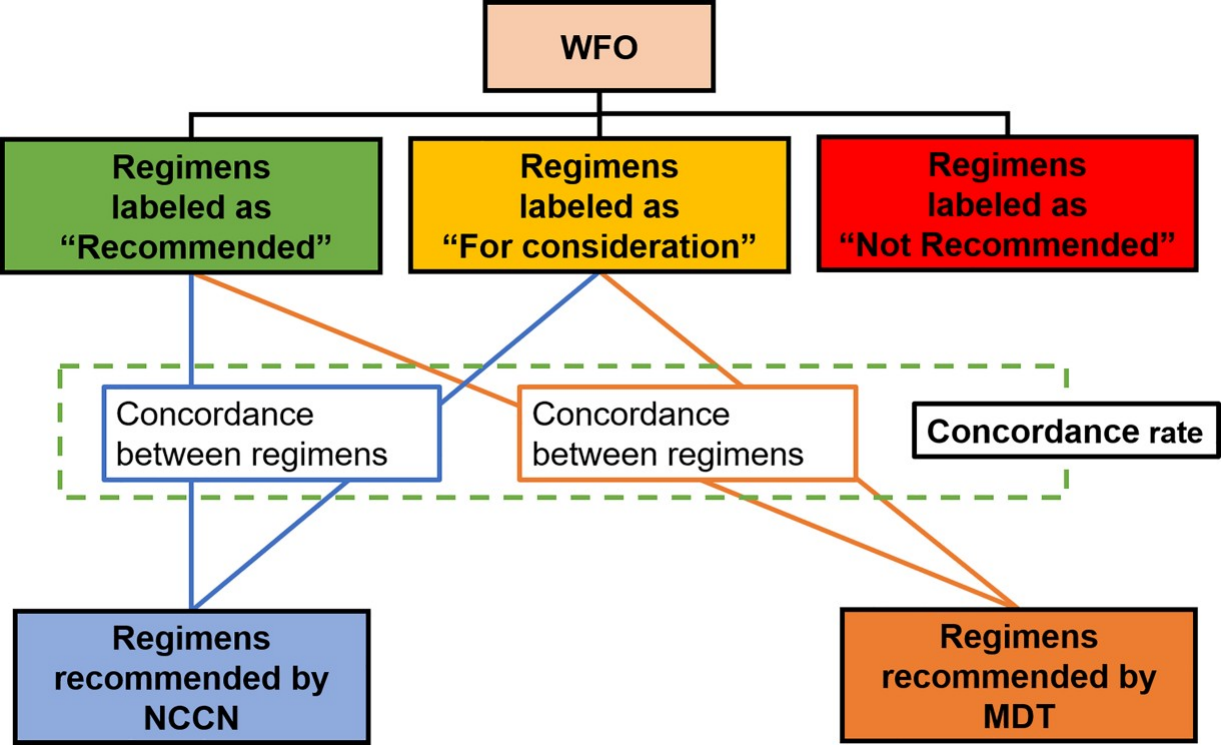
A diagram showing how the concordance between groups was measured.

The candidate therapeutic options were derived from the NCCN guidelines and the database of the Memorial Sloan Kettering Cancer Center (MSKCC), which was used to train the current version of WFO. The WFO gathers information on inclusion/exclusion criteria, drugs, comorbidities, contraindications, and MSKCC treatment preferences and also provides evidence for each treatment option by analyzing previously published studies, textbooks, and journals. To prioritize the chemotherapy options, the WFO uses Watson’s scoring algorithms, which are confidential. The WFO suggests the following three categories of treatment options: ‘Recommended’, ‘For consideration’, and ‘Not recommended’. We analyzed the concordance rates between the WFO ‘Recommended’ and/or ‘For consideration’ treatment options and the final therapeutic decision by the MDT (Fig 1).

### Statistical analyses

Statistical analyses were performed using SPSS for Windows version 23.0 (IBM Corporation, Armonk, NY, USA). Descriptive values were presented as means ± SD or numbers and percentages. To compare categorical variables between groups, the chi-squared test or Fisher’s exact test was used. To elucidate the agreements between the MDT and WFO regimens using the NCCN guidelines (NCCN guidelines for colon cancer, version 1.2017/ NCCN guidelines for rectal cancer, version 1.2017), regimens recommended by both WFO (including the ‘For consideration’ regimens) and the MDT were classified based on whether those regimens were concordant with the NCCN guidelines, by an experienced gastroenterologist. Agreements between groups were assessed using Cohen’s kappa coefficient. A *P*-value < 0.05 was considered statistically significant. To elucidate the factors for concordance between WFO-’Recommended’ regimens and the final decision made by the MDT, variables with a *P*-value < 0.2 in the univariate analysis were included in a forward stepwise multivariate logistic regression analysis. The regression analysis results were odds ratios and 95% confidence intervals (CIs) representing the probability of concordance in a given group compared with the reference group. Informed consent was not required for this type of study.

## Results

In this study, 69 patients who have presented to our hospital since the initiation of the multidisciplinary care service with WFO were included. The mean age of these patients was 62 years (range: 34–86 years), and more patients were male (47/69) than female (Table 1). Of the 69 patients, 51 (73.9%) were diagnosed with colon cancer, and all patients had good performance. Most patients had undergone surgery (n = 45, 65.2%) as a prior therapy. Of the 69 patients, 42 (60.9%) required adjuvant chemotherapy, 2 (2.9%) with rectal cancer required neoadjuvant chemotherapy, 23 (33.3%) received palliative chemotherapy, and 2 (2.9%) required surveillance only. The most common stage of the patients was IIIB (n = 27, 39.1%), followed by IV (n = 23, 33.3%). Mutations in Ras and EGFR were present in 26 (37.7%) and 51 (73.9%) patients, respectively. Regarding recurrence, seven (10.1%) patients had a history of recurrence before receiving the MDT service, and one (1.4%) experienced recurrence after the MDT service.

### Concordance rates between groups

**Fig 2:**
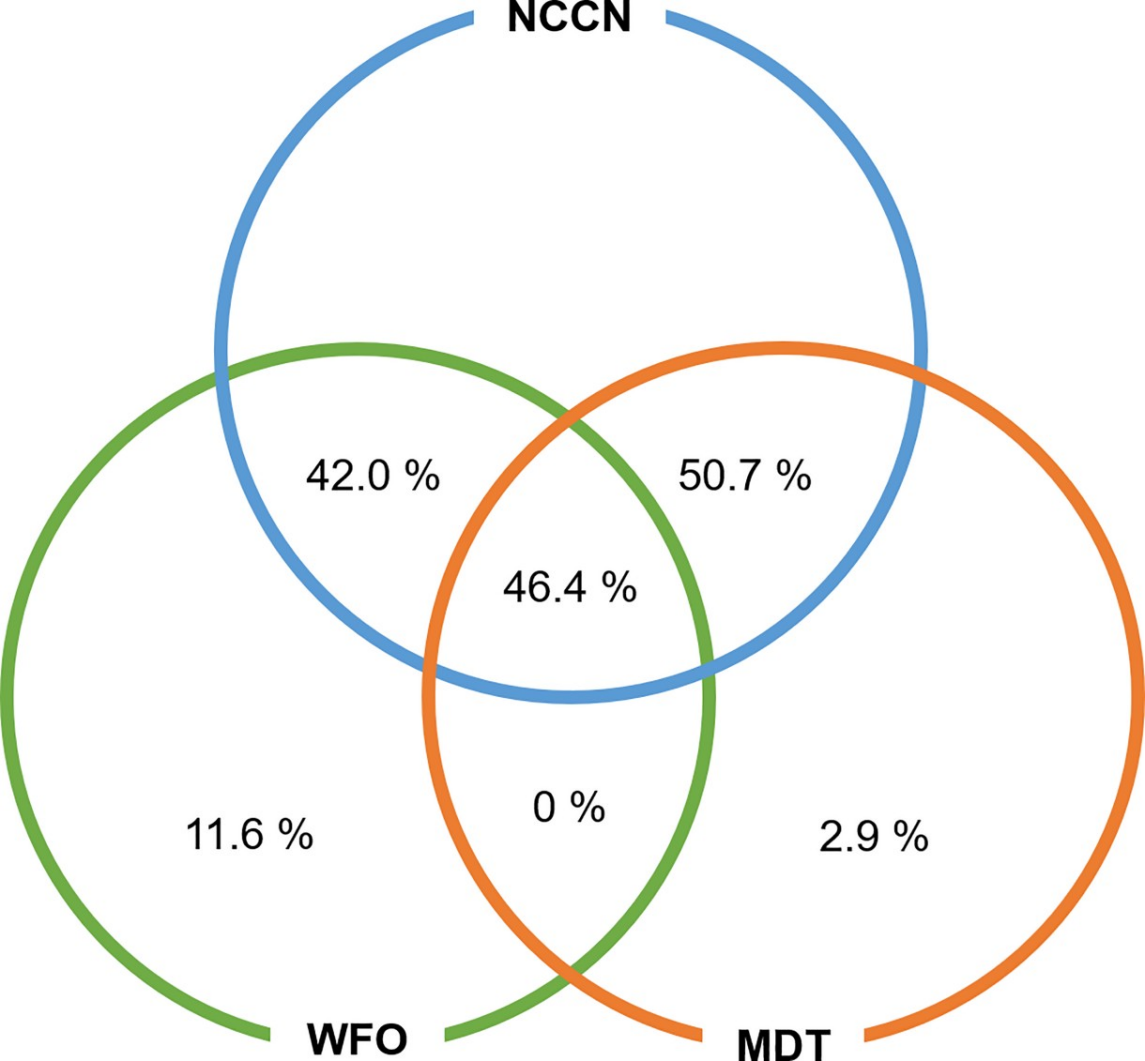
Diagram of the concordance rates. The WFO ‘Recommended’ treatment option and MDT recommendation.

**Fig 3:**
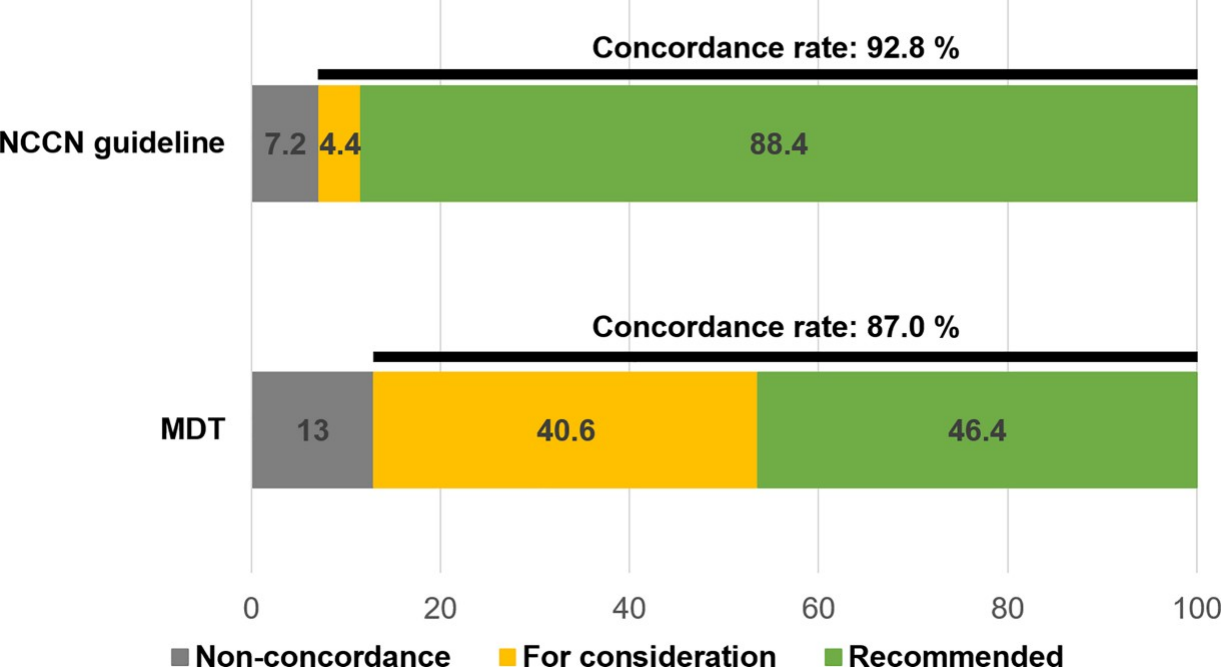
Concordance rates between WFO and the other groups.

The concordance rate between the MDT and NCCN guidelines was 97.1%, and that between the WFO and NCCN guidelines was 88.4%. (Fig 2) The extent of agreement between WFO and MDT recommendations based on the NCCN guidelines was tested by Cohen’s kappa statistic. The kappa value of 0.255 (*P* = 0.018) indicated fair agreement. The concordance rate between the chemotherapy options recommended by the MDT and WFO was 46.4%. After including the ‘For consideration’ treatment options determined by WFO, the concordance rate increased to 87.0%. (Table 2; Fig 3)

**Table 2:** The concordance rates between WFO- and MDT-recommended chemotherapy options.

	WFO (including ‘For consideration’)	MDT	NCCN
WFO (including ‘For consideration’)	NA	60 (87.0%)	64 (92.8%)
MDT	60 (87.0%)	NA	67 (97.1%)

**Fig 4:**
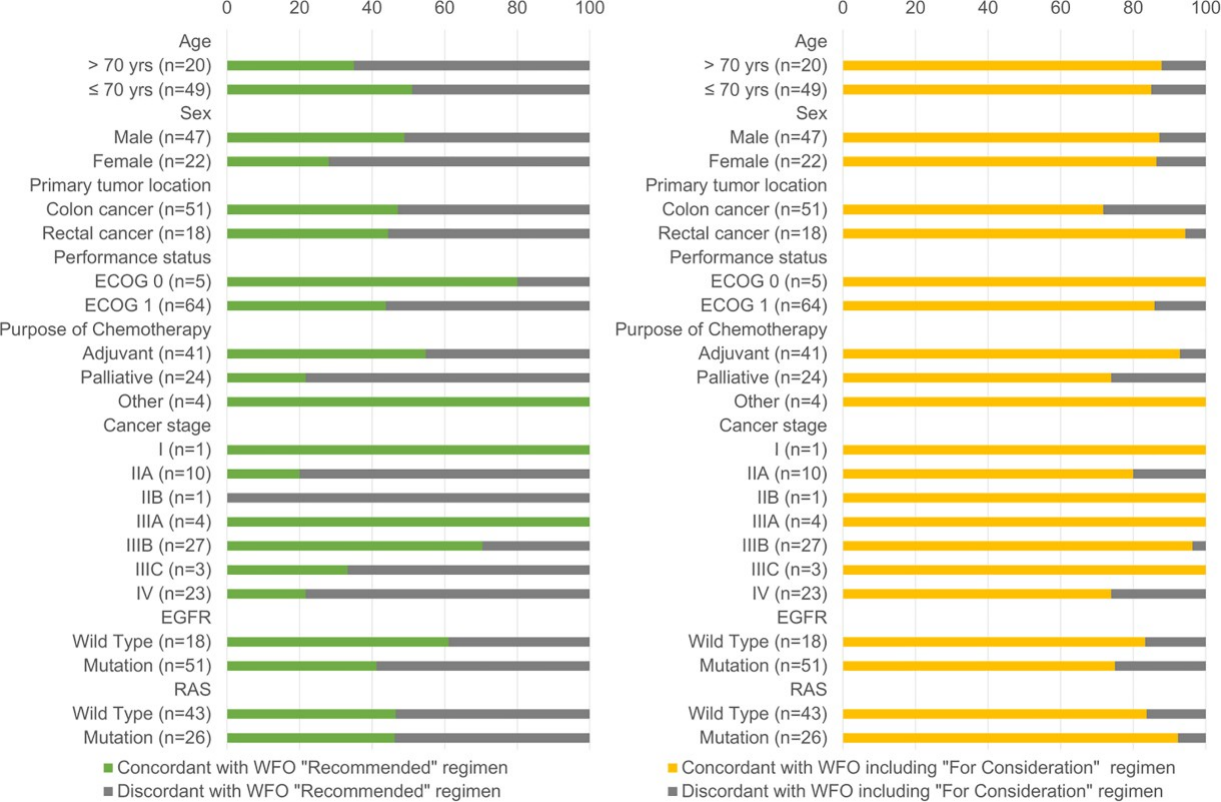
Comparison of concordance rates between WFO and MDT stratified by clinical factors.

Of the 69 patients, 51 (73.9%) were diagnosed with colon cancer and 18 (26.1%) with rectal cancer. The concordance rate of the chemotherapy options recommended by WFO versus the MDT was 47.1% for patients with colon cancer and 44.4% for patients with rectal cancer (*P*>0.999). However, in a comparison of the concordance rates among the chemotherapy regimens recommended by WFO (i.e., adjuvant chemotherapy, palliative chemotherapy, neoadjuvant chemotherapy, and surveillance), the concordance rate was significantly lower in patients who received palliative chemotherapy than in the other groups (*P* = 0.005; Fig 4).

Regarding cancer stage, the concordance rate between the WFO- and MDT-recommended chemotherapy options was significantly lower in patients with stage II, IIIC, or IV than in those with other stages (*P*<0.001; Fig 4). Among patients with colon cancer, the concordance rate between the WFO- and MDT-recommended chemotherapy options was relatively lower in those with stage II, IIIC, and IV disease (*P* = 0.051). The concordance rate between the WFO- and MDT-recommended chemotherapy options was significantly lower in patients with stage IV rectal cancer compared with the other stages (*P* = 0.004). When comparing older and < 70-year-old patients, there was no significant difference (*P* = 0.291; Fig 4).

In the multivariate analysis, which included performance status, when analyzing variables including type of chemotherapy (palliative vs. other), cancer stage, and EGFR mutation status, cancer stage (II, IIIC or IV) was the only factor that significantly influenced the concordance rate between the WFO (‘Recommended’ only) and MDT-determined regimens (OR: 0.092, 95% CI: 0.030–0.282) and between the WFO (‘For consideration’)- and MDT-determined regimens (OR: 0.117, 95% CI: 0.014–0.994)

## Discussion

Cognitive computing system or AI is increasingly being considered as a novel technology in healthcare. WFO is one such effort to develop a computing system to support clinical decision-making in medical practice. This study analyzed retrospective data to assess the efficacy and reliability of the current version of WFO for recommending treatment options for colorectal cancer patients who are candidates for systemic chemotherapy. The concordance rates between therapeutic plans determined by the MDT in the Republic of Korea and those determined by WFO and the possible factors related to non-concordance between the two groups were analyzed.

The concordance rate between the final recommendation by WFO (labeled as ‘Recommended’) and the NCCN guideline was 88.4%, and that between the recommendation by WFO and the final decision made by the MDT was relatively low (46.4%) despite the high concordance rate between the MDT decision and NCCN guideline (97.1%; S1 Dataset). However, the concordance rate between WFO and the MDT increased dramatically to 87.0% after including the ‘For consideration’ treatment options determined by WFO.

Currently, the NCCN guidelines suggest several possible chemotherapeutic regimens for colorectal cancer patients, among which physician choice is one such regimen for a specific patient. In clinical practice, there are many factors that can influence the physician’s final decision; these factors include the patient’s ethnicity, genetic information, general condition and preferences, the health insurance system, cost of treatment, and possible adverse events caused by particular chemotherapeutic agents. Based on our results, in general, WFO recommends chemotherapeutic regimens according to NCCN guidelines but has a distinct prioritizing algorithm that is different from that of the MDT in Korea. This is likely because WFO is not just a computing algorithm that summarizes the current NCCN guidelines but a prioritizing system that is based on database training with thousands of clinical practice cases from a tertiary medical center located in the USA (MSKCC).

We analyzed concordance rates according to age, sex, primary tumor location, performance status, purpose of chemotherapy, cancer stage, and genetic mutations. In the multivariate analysis, cancer stage was the only statistically significant factor that influenced the concordance rates between WFO and MDT. There are several possible explanations for this result. WFO prefers older regimens to those incorporating oxaliplatin [5–8] or bevacizumab [9–12]. In addition, FOLFIRI plus cetuximab is often recommended for patients with advanced or metastatic colon cancer harboring wild-type KRAS [13, 14]. However, WFO considers there to be little evidence for the FOLFIRI plus cetuximab regimen. WFO did not recommend regimens for which adverse events have been reported by multiple studies. In patients older than 70 years, WFO does not recommend oxaliplatin because of peripheral neuropathies [5, 15–17], or bevacizumab, which it advises to use with caution [11, 18]. In patients with stage IV rectal cancer, both FOLFIRI and FOLFOX have been used as palliative chemotherapies because of their low-grade (grade 1/2) toxicities and comparably good efficacies [19]. However, the WFO’s algorithms recommend FOLFIRI over FOLFOX for patients with stage IV rectal cancer.

Additionally, it seems that WFO does not recommend chemotherapy regimens with reportedly lower efficacies. For patients with stage II colon cancer with high-risk characteristics, the NCCN guidelines recommend adjuvant chemotherapy, including 5-FU-based regimens such as 5-FU plus leucovorin and FOLFOX [20]. The NCCN guidelines define high-risk characteristics as a poorly differentiated histology (excluding cancers with an MSI-H status), lymphatic/vascular invasion, bowel obstruction, less than 12 lymph nodes examined, localized perforation, and positive margins. Several studies showed that stage II MSI-H patients do not benefit from 5-FU-based adjuvant chemotherapy [21–24]. However, if patients with stage II colon cancer have MSI-H in addition to other high-risk factors, most clinicians may choose to prescribe adjuvant chemotherapy regimens, such as FOLFOX. Regardless of the presence of other high-risk factors, WFO recommended surveillance only for such patients.

Differences in the medical environment between the USA, where WFO was trained, and the Republic of Korea could be another possible explanation for the discrepancy. The national health insurance system in the Republic of Korea reimburses many chemotherapy regimens that are supported by the NCCN guideline. However, 5-FU-based chemotherapy for patients with MSI-high (MSI-H) stage IV disease are eligible for reimbursement despite that these patients may not benefit from 5-FU-based chemotherapy according to the NCCN guidelines. In patients with stage IV rectal cancer, both WFO and the MDT recommended regorafenib [25]. However, some patients received 5-FU-based chemotherapy, because regorafenib is expensive and not covered by the government in the Republic of Korea.

There are some limitations in this study. First, this was a retrospective study with a small sample size. Second, we used IBM WFO version 16.9, and different results might be obtained if another version of the WFO system or NCCN guidelines is used. Because cognitive computing systems and clinical guidelines continue to evolve as new data are generated, concordance rates might differ based on the version of the system. Up to date versions of the WFO and NCCN guidelines should be validated in further works. Third, the concordance rate is only an indication of the level of agreement between WFO and MDT and does not represent a clinical benefit of using cognitive computing systems, such as prolongation of overall survival or event-free survival. The concordance rate shows the potential possibility that a cognitive computing system can be used as a clinical assistant to help physicians make medical decisions. Further well-designed prospective studies are required to determine the clinical efficacy or utility of cognitive computing systems.

In conclusion, the concordance rate between chemotherapy regimens for colorectal cancer recommended by MDT and those recommended by WFO was 46.4%. After including the ‘For consideration’ option from WFO, the concordance rate increased to 88.4%. Further modifications and improvements of the WFO prioritizing algorithm may render WFO a useful clinical assistant for recommending treatment regimens.

## Supporting information

S1 Dataset All relevant data available. (XLSX)
